# Functional genomics of lung cancer progression reveals mechanism of metastasis suppressor function

**DOI:** 10.1186/1755-8166-7-S1-I9

**Published:** 2014-01-21

**Authors:** Ram Krishna Thakur, Vinod Kumar Yadav, Akinchan Kumar, Richa Basundra, Anirban Kar, Rashi Halder, Ankita Singh, Pankaj Kumar, Aradhita Baral, MJ Mahesh Kumar, Krishnendu Pal, Rajkumar Banerjee, Shantanu Chowdhury

**Affiliations:** 1 Proteomics and Structural Biology Unit; 2 G.N.R. Knowledge Centre for Genome Informatics, CSIRInstitute of Genomics and Integrative Biology, Delhi, India; 3 Animal House, CSIR-Centre for Cellular and Molecular Biology, Uppal Road, Hyderabad, India; 4 Division of Lipid Science and Technology, CSIR-Indian Institute of Chemical Technology, Hyderabad, India

## 

The mechanism of action of NME2, a widely accepted metastasis-suppressor gene, is poorly understood. Recently we found that NME2 directly regulates transcription of the *c-MYC* proto-oncogene. This prompted a genome-wide study to ascertain whether NME2 exerts its anti-metastatic action through transcriptional regulation. Chromatin-immunoprecipitation followed by massively parallel sequencing (ChIPseq) along with transcriptome profiling uncovered a network of genes involved in intercellular contact, focal adhesion and actin assembly under direct transcriptional control of NME2. In line with this, NME2-depleted cells displayed increased focal adhesion points and altered actin stress fiber organization. Our findings demonstrate that NME2 regulates transcription of a key focal adhesion factor vinculin and its localization within adhesion foci. NME2-depleted A549 lung cancer cells showed higher invasiveness in vitro and seeded more metastases in vivo. Consistent with these findings, expression of several NME2-transcriptional target genes related closely to advanced tumor stages with metastatic proclivity, and NME2 levels predicted patient survival.

